# Hypocretin/Orexin Peptides Excite Rat Neuroendocrine Dopamine Neurons through Orexin 2 Receptor-Mediated Activation of a Mixed Cation Current

**DOI:** 10.1038/srep41535

**Published:** 2017-02-01

**Authors:** David J. Lyons, Arash Hellysaz, Rachida Ammari, Christian Broberger

**Affiliations:** 1 Department of Neuroscience, Karolinska Institutet, Stockholm, Sweden

## Abstract

Hypocretin/Orexin (H/O) neurons of the lateral hypothalamus are compelling modulator candidates for the chronobiology of neuroendocrine output and, as a consequence, hormone release from the anterior pituitary. Here we investigate the effects of H/O peptides upon tuberoinfundibular dopamine (TIDA) neurons – cells which control, via inhibition, the pituitary secretion of prolactin. In whole cell recordings performed in male rat hypothalamic slices, application of H/O-A, as well as H/O-B, excited oscillating TIDA neurons, inducing a reversible depolarising switch from phasic to tonic discharge. The H/O-induced inward current underpinning this effect was post-synaptic (as it endured in the presence of tetrodotoxin), appeared to be carried by a Na^+^-dependent transient receptor potential-like channel (as it was blocked by 2-APB and was diminished by removal of extracellular Na^+^), and was a consequence of OX2R receptor activation (as it was blocked by the OX2R receptor antagonist TCS OX2 29, but not the OX1R receptor antagonist SB 334867). Application of the hormone, melatonin, failed to alter TIDA membrane potential or oscillatory activity. This first description of the electrophysiological effects of H/Os upon the TIDA network identifies cellular mechanisms that may contribute to the circadian rhythmicity of prolactin secretion.

Prolactin (Prl) is a functionally pleiotropic pituitary hormone that is involved in the regulation of numerous biological processes, including reproduction and energy balance^1^. Accordingly, both Prl secretion from the pituitary and the activity of tuberoinfundibular dopamine (TIDA) neurons – the hypothalamic neuroendocrine cells that regulate Prl release^2^ – are influenced by a range of physiological factors and events. Such circumstances include the estrous cycle^3^, mating^4^ and the suckling of offspring (see ref. 5).

These function-specific changes in Prl secretion are superimposed upon a daily rhythm of release, with Prl plasma levels in rats^6^,^7^ and humans^8^,^9^ exhibiting a peak in the late sleep phase. This underlying pattern of secretion persists in constant darkness but is abolished by constant light conditions in rats^10^,^11^ (as well as by ablation of the suprachiasmatic nucleus^7^,^12^,^13^). What cellular process drives this nychthemeral secretion? An answer may lie with the observation that TIDA neurons also exhibit a circadian pattern of activity, with their release of the Prl inhibiting factor, dopamine, being highest during waking and lowest during sleep^13^,^14^. Thus, it appears that sleep relevant factors may shape the circadian profile of Prl release by entraining the activity of TIDA neurons.

The hypothalamic neuropeptides hypocretin/orexin (H/O)-A and -B, are wakefulness-promoting modulators that play prominent roles in the regulation of sleep and metabolism^15^,^16^. Hypocretin/orexin-A and B are, respectively, 33 and 28 amino acid residues long, and in the brain are exclusively expressed in a discrete population of neurons found in the lateral hypothalamic area^15^,^16^. Hypocretin/orexin neurons project widely throughout the cerebral cortex, thalamus, hypothalamus and brainstem^17^,^18^ and the loss of these cells, or of H/O signalling, results in the sleep disorder narcolepsy in both humans^18^,^19^ and animals^20^,^21^. The H/O system is therefore believed to integrate the sleep wake cycle with other physiological processes. One manifestation of such a role is the powerful inhibition of Prl secretion observed following the central administration of H/Os^22^,^23^,^24^. Yet, the mechanistic underpinnings of this neuroendocrine modulation remain poorly understood. To investigate the possibility that H/O attenuates Prl release through actions on neuroendocrine dopamine cells, oscillating TIDA neurons – located in the dorsomedial aspect of the hypothalamic arcuate nucleus (Arc)^25^,^26^,^27^,^28^,^29^ – were patch clamped and the nature of their responsiveness to H/Os assessed.

## Materials and Methods

### Animals

Male Sprague-Dawley rats (Charles River), 22 to 30 days old, were housed with free access to standard chow and tap water in a temperature-controlled environment under 12/12 h light/dark conditions with lights on at 6 A.M. For this study of the Prl-inhibitory TIDA cells, we used male rats where inhibition is likely at its most active and to avoid the confounding influence from fluctuations in sex hormones, which are potent endogenous modulators of Prl secretion^30^. All animal experiments had received prior approval by the local ethical board, *Stockholms Norra Djurförsöksetiska Nämnd*, and were carried out in accordance with the European Communities Council Directive of 24 Nov. 1986 (86/609/EEC).

### Whole-Cell Recordings

For electrophysiological experiments rats (n = 27) were deeply anaesthetized with sodium pentobarbital and decapitated. The brain was rapidly removed and placed in an ice-cold and oxygenated (95%O_2_/5%CO_2_) ‘slicing’ solution containing (in mM) sucrose (214), KCl (2.0), NaH_2_PO_4_ (1.2), NaHCO_3_ (26), MgSO_4_ (1.3), CaCl_2_ (2.4), D-glucose (10). The meninges were gently removed, and the brain was blocked and glued to a vibratome (Leica) where 250 μm thick coronal slices of the hypothalamus containing the Arc were prepared. Slices were immediately transferred to artificial cerebrospinal fluid (aCSF) containing (in mM) NaCl (127), KCl (2.0), NaH_2_PO_4_ (1.2), NaHCO_3_ (26), MgCl_2_ (1.3), CaCl_2_ (2.4), D-glucose (10), in a continuously oxygenated holding chamber at 35 °C for a period of 25 min. Subsequently, slices were allowed to recover in ‘recording’ solution at room temperature for a minimum of 1 h before recording. For whole-cell recordings, slices were transferred to a submerged chamber and placed on an elevated grid that allows perfusion both above and below the slice. An Axioskop 2 FS Plus upright microscope (Carl Zeiss) was used for infrared - differential interference contrast visualization of cells. Recordings were performed at room temperature (22 °C) and slices were continuously perfused with oxygenated ‘recording’ solution (as above) at a rate of ca. 5 ml/min, unless otherwise described. All pharmacological compounds were bath applied.

Whole cell current- and voltage-clamp recordings were performed with pipettes (3–7 MΩ when filled with intracellular solution) made from borosilicate glass capillaries (World Precision Instruments) pulled on a P-97 Flaming/Brown micropipette puller (Sutter). The intracellular recording solution contained (in mM) K-gluconate (140), KCl (10), HEPES (10), EGTA (1), Na_2_ATP (2), pH 7.3 (with KOH). The concentration used for tetrodotoxin (TTX) was 500 nM. In voltage clamp experiments, unless otherwise stated, the holding potential was −60 mV. In experiments that required the inhibition of Na^+^-dependent conductances a ‘Zero Na^+^’ extracellular solution was prepared where NaCl was substituted with an equimolar concentration of TRIS-HCl. Recordings were performed using a Multiclamp 700B amplifier and pClamp9 software (Molecular Devices). Slow and fast capacitative components were automatically compensated for. Access resistance was monitored throughout the experiments, and neurons in which the series resistance was >25 MΩ or changed >15% were excluded from the statistics. Liquid junction potential was 16.4 mV and not compensated. The recorded current was sampled at 10 kHz and filtered at 2 kHz unless otherwise stated.

### Immunofluorescence and image analysis

Male 6–10 weeks old Sprague Dawley rats (n = 5), were deeply anesthetized and perfused with a fixative containing 4% paraformaldehyde and 0.2% picric acid according to Zamboni and deMartino^31^. Fourteen μm thick sections spanning the entire rostro-caudal length of the Arc were processed for immunofluorescence using the tyramide signal amplification (TSA) Plus protocol (Perkin Elmer) as previously described^32^. Briefly, sections were incubated with primary anti-tyrosine hydroxylase (TH) immunoglobulin (1:1,000; mouse monoclonal; MAB318; Millipore) combined with anti-H/O antiserum (1:10,000; raised in rabbit; gift from Drs. E. Mignot and K. Eriksson) at 4 °C for 16 hours. Sections were then pre-incubated with TNB blocking reagent as supplied with TSA Plus kit (Perkin Elmer) for 30 min, and incubated for 60 min with horseradish-peroxidase-conjugated swine anti-rabbit immunoglobulin (1:500 in TNB buffer; Dako) mixed with donkey anti-mouse antiserum conjugated with Alexa-594 (Life Technologies). The sections were next incubated for 10 min with tyramide-conjugated fluorescein (1:500) and subsequently mounted with anti-fading agent (2.5% DABCO, Sigma) diluted in glycerol. Three tissue sections per animal, spaced at regular intervals throughout the Arc were investigated with confocal microscopy using an Olympus FV1000 microscope (Tokyo, Japan). For each brain section and hemisphere, high magnification confocal stacks were acquired from the dm Arc. Image stacks were processed and analyzed with BitPlane Imaris software. Close appositions were assigned manually by investigating the stack’s 3D projections, and confirmed by investigating single optical sections in the xy-, xz- and yz-planes.

### Statistical Analysis and Reagents

Data analysis was performed with OriginPro8.5 (OriginLab) and Clampfit 9 (Molecular Devices) software. Statistical significance was set at P < 0.05 and determined using the stated statistical test (*P < 0.05, **P < 0.01, ***P < 0.001, ns = non-significant). To determine the H/O-A induced current in the presence of TTX frequency distribution histograms were plotted for ten Sec of control, H/O-A and wash traces, with Gaussian fits performed on the individual and pooled distributions. The mean difference between the Gaussian peaks for control and H/O-A was used as the value for the H/O-A induced current.

All reagents were purchased from Sigma Pharmaceuticals with the exception of TTX which was purchased from Alomone Labs, H/O-A and –B which were purchased from Bachem and SB 334867, Ala^11^D-Leu^15^-orexin B and TCS OX2 29 which were purchased from Tocris Bioscience.

## Results

### TIDA neurons are innervated by H/O-immunoreactive fibers

**Figure 1:**
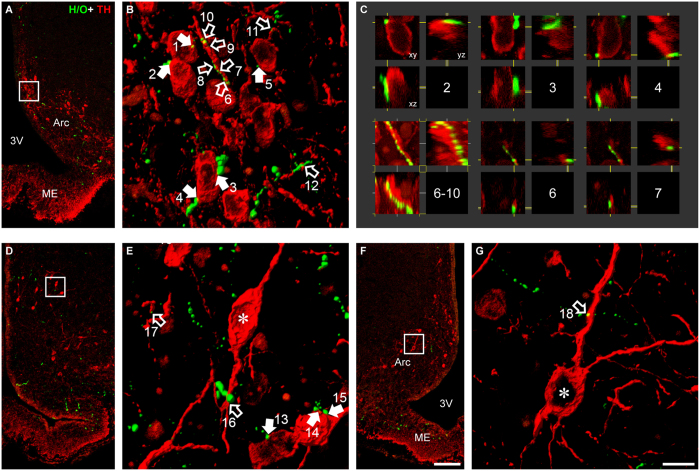
Hypocretin/orexin terminals form close appositions to TIDA neurons. Low power confocal micrographs (**A**,**D**,**F**) from three different rat Arc sections and levels processed for immunofluorescence for tyrosine hydroxylase (TH; red) and H/O (green). Orthogonal normal shadings of confocal stacks in (**B**,**E**,**G**) represent regions within squares in (**A**,**D**,**F**) respectively. Note close appositions between H/O-immunoreactive (-ir) terminals and TH-ir cell somata (**B**,**E**; filled arrows) and dendrites (**B**, **E**,**G**; empty arrows). All close appositions are confirmed in 3D with single optical sections in the xy-, xz- and yz-plane (examples from arrows in **B** represented in **C** with corresponding number). 3V, third ventricle; Arc, arcuate nucleus; ME, median eminence. Scale bar in (**F**) = 100 μm for (**A**,**D**,**F**) and in (**G**) = 10 μm for (**B**,**E**,**G**).

The H/O neurons of the lateral hypothalamus project widely throughout the brain, innervating numerous structures – including the Arc^17^,^33^,^34^. It remains unknown, however, to what extent this innervation includes the dmArc and TIDA neurons. Double-label immunofluorescence staining of H/O and tyrosine hydroxylase (TH) – the rate-limiting enzyme in monoamine biosynthesis – was performed to address this issue (Fig. 1). Hypocretin/Orexin-immunoreactive (-ir) fibers were observed in all three sectors of the Arc (the dorsomedial, ventromedial and ventrolateral^35^). Only scattered axon terminals were seen in the median eminence and then exclusively in the internal layer, separate from the TH-ir terminals in the external layer. TH-ir neurons clustered in the dmArc were observed to form close appositions on both cell somata and proximal dendrites. Such contacts could be seen throughout the rostrocaudal extension of the dmArc. Individual appositions were verified in confocal microscope images in x-, y-, and z-planes to ensure that they did not simply represent superimpositions upon TH-ir elements in the coronal plane. Thus, there appears to exist an anatomical substrate for direct H/O-TIDA interactions. We next explored this possibility using whole-cell patch clamp recordings.

### Hypocretin/orexin A and B excite oscillating TIDA neurons replacing phasic discharge with tonic firing via a post synaptic mechanism

**Figure 2:**
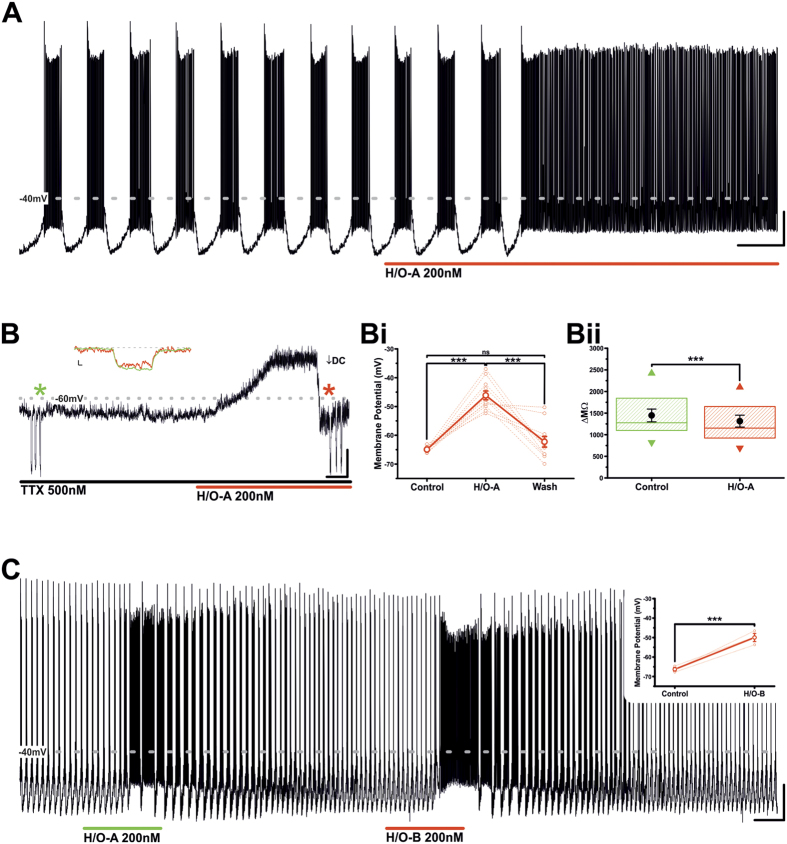
Hypocretin/Orexin-A and B excite TIDA neurons. (**A**) Current clamp recording of an oscillating TIDA neuron. Application of H/O-A results in excitation and a switch from phasic to tonic firing. Scale bar 20 mV/20Sec. (**B**) Current clamp recording of a TIDA neuron in the continuous presence of TTX, applied to abolish oscillation to achieve a stable baseline. Prior to application neuron was held at −65 mV with negative DC current injection and input resistance assessed with test pulses of −20pA (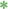). The H/O-A induced depolarisation endured in the presence of TTX. To compare input resistance at peak of response with that in control, membrane potential was returned to −65 mV with additional negative DC current injection (↓) and identical test pulses reapplied (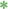). Inset are examples of control (green) and response (red) test pulses superimposed. Scale bar 10 mV/10 Sec; inset 5 mV/50 mSec. (**Bi)** Population data from recordings as illustrated in (**B**). Application of H/O-A causes a reversible and significant depolarisation of resting membrane potential. The mean membrane potential in control, H/O-A and wash is represented by the solid line. Raw data used to produce means shown as dashed lines (n = 11; ***P < 0.001; ns; ANOVA repeated measures with *post-hoc* Tukey test). (**Bii)** The H/O-A induced depolarisation is associated with a significant decrease in input resistance (n = 11; ***P < 0.001; paired t-test). Boxes represent 25, 50 and 75 percentile with superimposed Mean ± SEM and maximum and minimum values. (**C)** Current clamp recording of a TIDA neuron exposed to H/O-A and, following wash and return to baseline conditions, H/O-B. Note similar reversible depolarising response to both peptides. Scale bar 20 mV/2 min. Inset – in the presence of TTX, H/O-B application causes a significant depolarisation of membrane potential. The mean membrane potential in control and H/O-B is represented by the solid line. Raw data used to produce means shown as dashed lines (n = 3; ***P < 0.001; paired t-test).

Earlier work has demonstrated that in the rat, TIDA neurons can be reliably identified *in vitro* on the basis of their electrophysiological properties^2^,^25^,^26^. These features include a robust membrane potential oscillation that is synchronised between neurons, such that TIDA cells alternate rhythmically between depolarised UP states crowned by action potentials and hyperpolarised DOWN states during which discharge is absent (Fig. 2A).

Bath application (90–120 seconds) of H/O-A (200 nM) consistently resulted in the depolarisation of TIDA neurons and the replacement of phasic discharge with tonic firing (Fig. 2A; n = 12/12; 100%), transient effects readily reversible upon wash of drug from the recording chamber. To evaluate the possibility that these effects are mediated by direct, postsynaptic actions of H/O-A on TIDA neurons, action potential-dependent pre-synaptic influences were blocked by bath application of the voltage-gated Na^+^ channel antagonist TTX (500 nM), a compound that also abolishes the TIDA oscillation, likely by its inhibitory effect on persistent Na^+^ currents^25^ (unpublished observations). In the presence of TTX, and from a membrane potential of ≈−65 mV, application of H/O-A induced a transient membrane potential depolarisation of 18.8 ± 1.6 mV (Fig. 2B,Bi; n = 11; P < 0.001; ANOVA; this value was used as a control H/O-A response for comparison with subsequent current clamp tests). The H/O-A -induced excitation was associated with a small, but significant, reduction in input resistance (Fig. 2B,Bii; n = 11; P < 0.001; t-test paired). Collectively, these results are consistent with the activation of a post-synaptic conductance.

While H/O-A activates both the H/O type 1 (OX1R) and the H/O type 2 (OX2R) receptors with equal potency, the H/O-B peptide exhibits much greater affinity to the OX2R than the OX1R^16^. Accordingly, we also applied H/O-B (200 nM) to our preparation, which similarly excited TIDA neurons and induced a comparable shift from phasic to tonic firing (Fig. 2C; n = 5/5; 100%). The H/O-B effect also proved to be post-synaptic, as in the presence of TTX H/O-B induced a depolarisation of 16.3 ± 1.9 mV (Fig. 2C inset; n = 3; P < 0.001; paired t-test), a value statistically indistinguishable from that of H/O-A (18.8 ± 1.6 mV *vs* 16.3 ± 1.9 mV; P > 0.05; unpaired t-test). These data provided the first indication of the involvement of the OX2R in these effects (see further below).

### Hypocretin/orexin A activates an inward current

**Figure 3:**
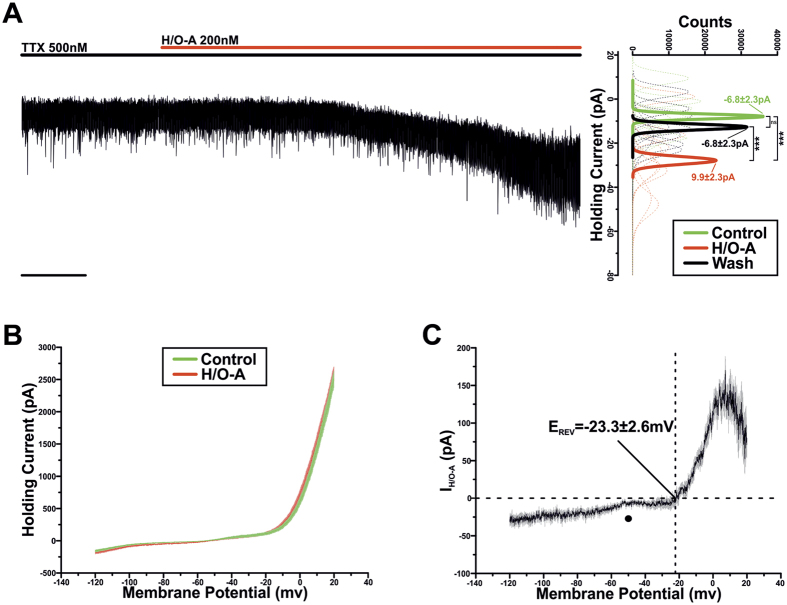
Hypocretin/Orexin-A activates an inward current. (**A)** Voltage clamp recording of a TIDA neuron in the presence of TTX applied to abolish oscillation and achieve a stable baseline. Application of H/O-A results in an inward current (Scale bar 20 Sec). To the right, sharing its y-axis with the raw trace, are Gaussian fits of averaged (solid lines) holding current frequency distributions in control (green), H/O-A (red) and wash (black). Raw data used to produce averages shown as dashed lines (n = 9; ***P < 0.001; ANOVA repeated measures with *post-hoc* Tukey test). (**B)** Averaged voltage clamp ramps (n = 9) acquired in control (green) and at the peak of response (red). Line = mean; shading = SEM. (**C**) H/O-A –induced current (I_H/O-A_) obtained by the digital subtraction of the traces displayed in (**B**). Note the negative slope conductance at −50 mV (●) and reversal at −23.3 ± 2.6 mV. Line = mean; shading = SEM.

To gain insight into the post-synaptic mechanisms underpinning this H/O-induced excitation, TIDA neurons were recorded in voltage clamp and in the presence of TTX (500 nM). Bath application of H/O-A (200 nM) induced a reversible inward current of −19.8 ± 3.1pA (Fig. 3A; n = 9; P < 0.001; ANOVA; this value was used as a control H/O-A response for comparison with subsequent voltage clamp tests).

The current-voltage relationship of the H/O-A -induced current (I_H/O-A_) was examined by voltage clamp ramps, which drove membrane potential from −120 mV to 20 mV at a rate of 45.7 mV.Sec^−1^ (Fig. 3B; n = 9). The digital subtraction of the ramp in control from the ramp at the peak of the response revealed I_H/O-A_ to have a negative slope conductance at approximately −50 mV and to reverse at −23.3 ± 2.6 mV (Fig. 3C; n = 9). These properties indicate that I_H/O-A_ is mediated by the activation of a mixed cationic conductance.

**Figure 4:**
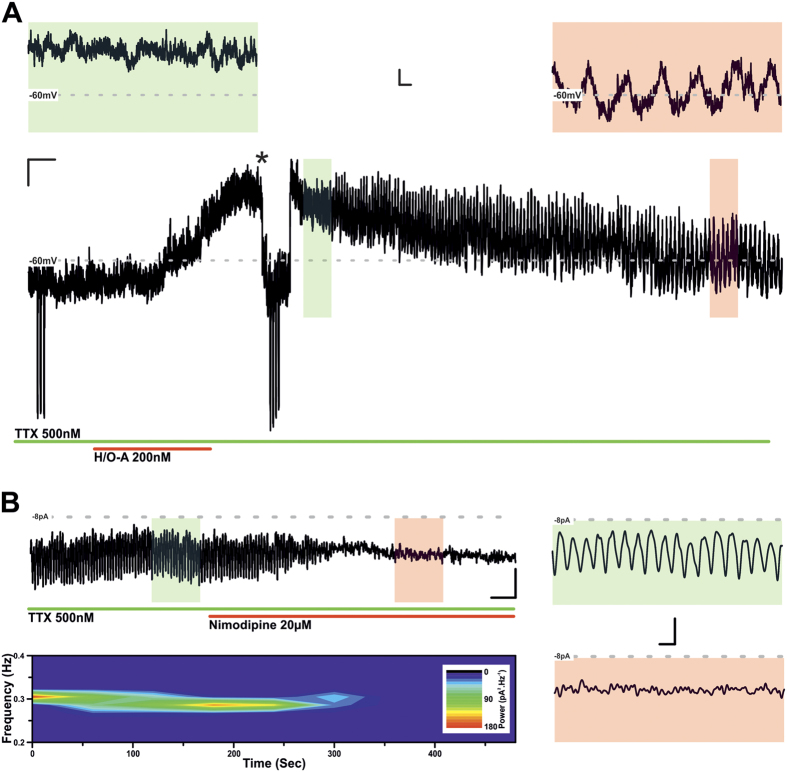
Hypocretin/Orexin-A activates a TTX-insensitive L-type dependent membrane potential oscillation. (**A**) Current clamp recording of an oscillating TIDA neuron in the continuous presence of TTX. Prior to application of H/O-A, neuron was held at −65 mV with negative DC current injection and input resistance assessed with test pulses of −20pA. Application of H/O-A induced a depolarisation as in (Fig. 2B). However, in 3/9 (33%) of neurons tested, following peak depolarisation (green inset) H/O-A application induced a TTX insensitive membrane potential oscillation with late onset (red inset). Scale bar 5 mV/20 Sec; inset 4 mV/1 Sec. (**B**) Voltage clamp recording of a TIDA neuron in the continuous presence of TTX and following the application of H/O-A. Trace is filtered at 1 Hz with associated spectrogram shown underneath (Scale bar 4pA/30 Sec). The H/O-A induced, late onset TTX-insensitive oscillation, was abolished by the application of L-type Ca^2+^ channel blocker nimodipine (3/3, 100%). Shaded areas inset correspond to control (green) and nimodipine (red). Scale bar 4pA/30 Sec; 4pA/4 Sec.

In a proportion (n = 3/9; 33%) of TIDA neurons application of H/O-A, in addition to the mixed cationic conductance, also generated a slow onset, TTX-insensitive oscillation (Fig. 4A). This rhythmical activity was characterised by a frequency of 0.3 ± 0.06 Hz (n = 3) and an amplitude of 6.2 ± 1.1pA; (n = 3), and manifested itself following the peak H/O-A induced response. (This H/O-generated, TTX-*insensitive* oscillation is significantly faster and of lower amplitude than the default, TTX-*sensitive* TIDA oscillation recorded at the same temperature (frequency: 0.05 ± 0.002 Hz; amplitude: 22.7 ± 2.9pA; n = 9)). The fast oscillation appeared to be mediated by an L-type Ca^+^ current as it was abolished by the application of the L-type antagonist, nimodipine (20 μM; Fig. 4B; n = 3/3).

### Hypocretin/orexin A -induced mixed cationic current is Na^+^-dependent and inhibited by the TRP channel blocker 2-APB

**Figure 5:**
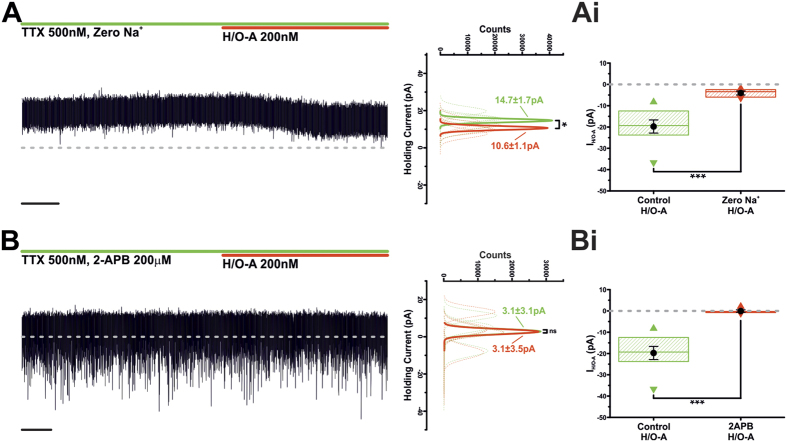
I_H/O-A_ is mediated by a Na^+^-dominated mixed cationic current. (**A**) Voltage clamp recording of an oscillating TIDA neuron in the presence of TTX and ‘Zero Na^+^’ recording solution. Application of H/O-A induced a small, yet significant, inward current (Scale bar 20 Sec). To the right, sharing its y-axis with the raw trace, are Gaussian fits of averaged (solid lines) holding current frequency distributions in control (green) and H/O-A (red). Raw data used to produce averages shown as dashed lines (n = 5; *P < 0.05; paired t-test). (**Ai)** Application of H/O-A in ‘Zero Na^+^’ recording solution results in a significant reduction in I_H/O-A_ when compared to control (Control, n = 9; ‘zero Na^+^’, n = 5; ***P < 0.001; unpaired t-test). Box plot organised as Fig. 2Bii. (**B)** Voltage clamp recording of a TIDA neuron in the presence of TTX and 2-APB (200 μM). Application of H/O-A failed to induce an inward current (Scale bar 20 Sec). To the right, sharing its y-axis with the raw trace, are Gaussian fits of averaged (solid lines) holding current frequency distributions in control (green) and H/O-A (red). Raw data used to produce averages shown as dashed lines (n = 5; ns; paired t-test). (**Bi)** Application H/O-A in the presence of 2-APB results in an abolishment of I_H/O-A_ (Control, n = 9; 2-APB, n = 5; ***P < 0.001; unpaired t-test). Box plot organised as Fig. 2Bii.

Mixed cationic currents can be carried by different positively charged ions, but are often dominated by Na^+^, a phenomenon previously reported both in TIDA neurons^26^,^27^ and in neuronal responses to H/O-A^36^. To test the role of Na^+^ ions in I_H/O-A_, H/O-A was applied in the presence of recording solution in which NaCl had been substituted with an equimolar amount of TRIS-HCl. Under these conditions application of H/O-A produced an inward current of only −4.0 ± 0.9pA (Fig. 5A; n = 5; P < 0.05; paired t-test), a value statistically different from control (Fig. 5Ai; P < 0.001 *vs* control; t-test unpaired). As I_H/O-A_ reverses near −20 mV, exhibits a negative slope conductance around −50 mV and is Na^+^ dominated, we suspected the involvement of a canonical transient receptor potential (TRPC) channel^26^,^37^. This possibility was evaluated pharmacologically, applying H/O-A in the presence of TTX and 2-aminoethoxydiphenylborate (2-APB; 200 μM) – a potent blocker of TRPC1, TRPC3, TRPC4, TRPC5 and TRPC6^38^ – which completely abolished the H/O induced effect (Fig. 5B). Under these conditions I_H/O-A_ was a statistically insignificant 0.08 ± 0.55pA (n = 5; ns; paired t-test), a value distinct from control (Fig. 5Bi; P < 0.001 *vs* control; unpaired t-test).

### Hypocretin/Orexin induced excitation most likely mediated by OX2 receptors

**Figure 6:**
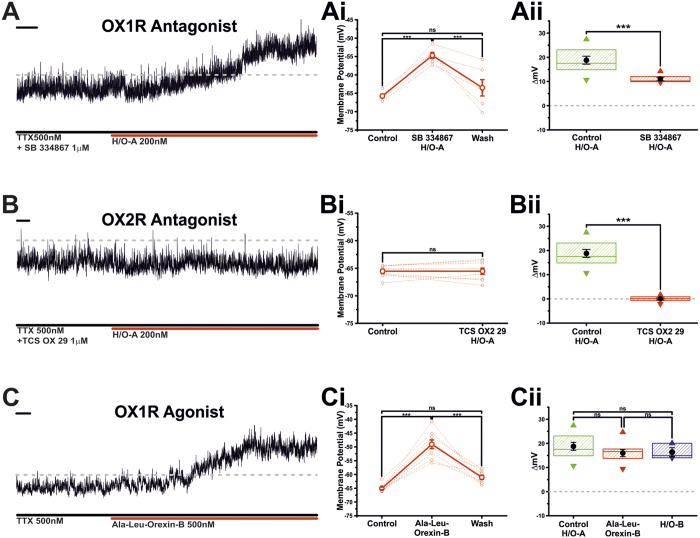
I_H/O-A_ is blocked by OX2R but not OX1R antagonists. (**A)** Current clamp recording of the response to H/O-A of a TIDA neuron in the continuous presence of TTX and the OX1R antagonist SB334867 (1 μM). Scale bar 20 Sec. (**Ai)** Sharing its y-axis with the raw trace in A, is the mean (solid line) membrane potential in control, H/O-A + SB334867 and wash. Raw data used to produce averages shown as dashed lines (n = 6; ***P < 0.001; ANOVA repeated measures with *post-hoc* Tukey test). (**Aii)** Blockade of the OX1R receptor with SB334867 failed to abolish H/O-A induced depolarisation, however, under these conditions the resultant depolarisation was significantly different to that of control (Control n = 11; H/O-A in SB334867 n = 6; ***P < 0.001; unpaired t-test). Box plot organised as Fig. 2Bii. (**B)** Current clamp recording of the response of a TIDA neuron to H/O-A in the presence of TTX and the selective OX2R Antagonist TCS OX2 29 (1 μM) (Scale bar 10 Sec). (**Bi)** Sharing its y-axis with the raw trace in B is the mean (solid line) membrane potential in control and H/O-A + TCS OX2 29. Raw data used to produce averages shown as dashed lines (n = 6; P > 0.05; paired t-test). (**Bii)** The presence of OX2R antagonist TCS OX2 29 completely abolished TIDA neurons response to H/O-A, an effect statistically different from the H/O-A induced depolarisation under control conditions (Control n = 11; TCS OX2 29 n = 9; ***P < 0.001; unpaired t-test). Box plot organised as Fig. 2Bii. (**C)** Current clamp recording of the response of a TIDA neuron in the continuous presence of TTX to selective OX2R agonist Ala-Leu-Orexin-B (500 nM) (Scale bar 10 Sec). (**Ci**) Sharing its y-axis with the raw trace in C, is the mean (solid line) membrane potential in control, Ala-Leu-Orexin-B and wash. Raw data used to produce averages shown as dashed lines (n = 6; ***P < 0.001; ANOVA repeated measures with *post-hoc* Tukey test). (**Cii)** Application of Ala-Leu-Orexin-B induced a depolarisation that was statistically indistinguishable from the H/O-A and H/O-B induced depolarisations under control conditions (Control n = 11; Leu-Ala-Orexin-B n = 9; H/O-B n = 3; P > 0.05; ANOVA with *post-hoc* Tukey test). Box plot organised as Fig. 2Bii.

The H/Os influence cellular physiology through their interaction with their cognate receptors, OX1R and OX2R^16^. To investigate the receptor responsible for the H/O-induced changes in TIDA electrophysiology we took advantage of three pharmacological tools; the OX1R antagonist SB 334867^39^, the OX2R antagonist TCS OX2 29^40^ and the OX2R agonist Ala^11^-D-Leu^15^Orexin-B^41^. Application of H/O-A in the continuous presence of SB 334867 (1 μM) did not block the H/O-A–induced depolarisation (Fig. 6A and Ai; n = 6; P < 0.001; paired t-test). The resultant depolarisation was, however, statistically different from that induced by H/O-A in the absence of the OX1R antagonist (Fig. 6Aii P < 0.001 *vs* control; unpaired t-test). In contrast, co-application of H/O-A with the highly selective OX2R antagonist TCS OX2 29 (1 μM) completely abolished the H/O-A -induced depolarisation (Fig. 6B and Bi; n = 9; ns; unpaired t-test). Furthermore, the highly selective OX2R agonist, Ala^11^-D-Leu^15^Orexin-B (500 nM), induced a large depolarisation of 16.0 ± 0.5 mV (Fig. 6C and Ci; n = 9; P < 0.001; paired t-test), a value that proved indistinguishable from both control H/O-A and H/O-B (Fig. 6Cii; ns; ANOVA). Collectively these pharmacological data indicate that H/O-A exerts its electrophysiological effects upon TIDA neurons via OX2R.

### TIDA neurons do not respond to melatonin

**Figure 7:**
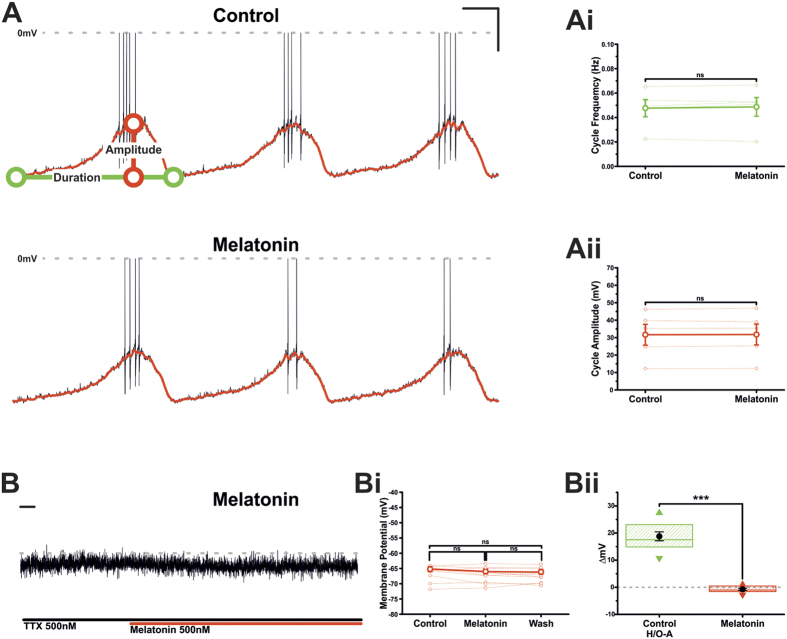
Melatonin fails to excite TIDA neurons. (**A)** Current clamp recording of an oscillating TIDA neuron in control and the presence of melatonin (500 nM). Scale bar 20 mV/4 Sec. (**Ai)** Application of melatonin failed to result in a significant change in oscillation cycle frequency. The mean value is represented by the solid line. Raw data used to produce means shown as dashed lines (n = 5; ns; paired t-test). (**Aii)** Application of melatonin failed to result in a significant change in oscillation cycle amplitude. The mean value is represented by the solid line. Raw data used to produce means shown as dashed lines (n = 5; ns; paired t-test). (**B)** Current clamp recording of a TIDA neuron in the continuous presence of TTX to abolish oscillation and achieve a stable baseline. Membrane potential remained unchanged after application of melatonin. Scale bar 20 Sec. (**Bi)** Sharing its y-axis with the raw trace in B, is the mean (solid line) membrane potential in control, melatonin and wash. Raw data used to produce averages shown as dashed lines (n = 10; ns; ANOVA repeated measures with *post-hoc* Tukey test). (**Bii)** Application of the melatonin failed to result in a significant change to TIDA neuron membrane potential, a response significantly different to that of control H/O (Control n = 11; Melatonin n = 10; ***unpaired t-test). Box plot organised as Fig. 2Bii.

Another major determinant of circadian rhythmicity is the pineal hormone, melatonin. Melatonin contributes to timing a range of physiological processes, including several neuroendocrine axes (see ref. 42). For Prl secretion, both stimulatory^43^ and inhibitory^44^ actions have been described upon systemic melatonin administration, seemingly contradictory effects that may reflect a role for melatonin in imposing *seasonal*, rather than daily, rhythms in the lactotrophic system (see ref. 45). In our final experiments, we addressed if melatonin affects the electrical behaviour of TIDA neurons. To investigate this, melatonin (500 nM) was bath applied to TIDA neurons. Melatonin application did not alter TIDA oscillation dynamics, leaving oscillation frequency (control 0.047 ± 0.007 Hz *vs* melatonin 0.049 ± 0.008 Hz; Fig. 7A–Aii; n = 5; ns; ANOVA) and amplitude (control 31.6 ± 6.0 mV *vs* melatonin 31.8 ± 6.0 mV; Fig. 7A–Aii; n = 5; ns; ANOVA) unchanged. In the presence of TTX and from a membrane potential of ≈−65 mV, application of melatonin failed to induce a change in membrane potential (control −66.4 ± 0.8 mV *vs* Melatonin −67.1 ± 0.8 mV; Fig. 7B–Bi; n = 10; ns; ANOVA), a response distinct from that observed with H/O-A (Fig. 7Bii; −0.7 ± 0.4 mV; n = 10; P < 0.001; t-test unpaired). These data suggest that H/Os, but not melatonin, function as primary chronobiological factors directly affecting the temporal patterning of TIDA activity.

## Discussion

In this study we investigated the effects of H/O peptides upon the electrical activity of TIDA neurons. We found that both H/O-A and -B excite TIDA neurons by inducing a depolarising switch from phasic discharge to tonic firing, a response that was frequently associated with a TTX-insensitive L-type Ca^2+^ channel-dependent oscillation. While earlier work has shown that centrally administered H/O can potently lower plasma Prl levels^22^,^23^,^24^, and has suggested that this may be relayed in part (but not fully) through dopaminergic actions^23^, the site of action and mechanism for this neuroendocrine modulation has remained elusive. As neuroendocrine dopamine exerts a powerful inhibitory influence on pituitary lactotrophs (see ref. 2), the excitation of TIDA neurons by H/O shown here identifies where and how H/O may suppress Prl release.

This excitatory effect is mediated by the activation of post-synaptic OX2R receptors, as it endures in the presence of TTX, can be elicited by both H/O-A and H/O-B (in agreement with actions on plasma Prl levels^24^) and is completely blocked by the OX2R antagonist TCS OX2 29, while being only partially inhibited by the OX1R antagonist SB 334867. The dominant role of OX2R in this excitatory response is further underscored by the depolarising effect of Ala^11^-D-Leu^15^Orexin-B – an OX2R selective agonist – which proved to be indistinguishable to that of H/O-A.

Neuronal excitation as a consequence of H/O receptor activation has been shown to occur in many neuronal populations and to be underpinned by an assortment of electrophysiological mechanisms. For example, H/Os cause excitation via closure of a K^+^ conductance in thalamic neurons^46^,^47^, sublayer 6b cortical neurons^48^ and layer 2–3 pyramidal cells^36^. In histaminergic neurons of the tuberomammillary nucleus^49^ and both pro-opiomelanocortin^50^ and GABA-ergic neurons of the Arc^51^ H/O causes excitation via the activation of a Na^+^/Ca^2+^ exchanger. In TIDA neurons the H/O effect is here shown to be underpinned by the activation of a mixed cation current, a mechanism shared with H/O-induced excitation in serotonin neurons of the dorsal raphe^52^,^53^, area postrema neurons^54^ and cholinergic neurons of the laterodorsal tegmentum^55^,^56^. In the latter example the H/O response is – like the TIDA effect – associated with the augmentation of L-type Ca^2+^ currents. Importantly, in addition to H/O, other Prl-inhibiting factors – such as TRH^25^ (when acting centrally) and oxytocin^27^, as well as Prl itself^26^ – induce excitation via the activation of a mixed cation current. It therefore appears that the modulation of these currents constitutes a key regulatory axis, or point of convergence, for factors controlling the output of the TIDA network.

Hormone secreting cells of the anterior pituitary are regulated primarily by tropic factors, substances secreted by hypothalamic neuroendocrine cells which then reach the pituitary via the portal circulation^57^. As H/O receptors have been localised in relevant neuronal populations^50^ and its intracerebroventricular administration has been shown to influence the secretion of pituitary hormones – including Prl^24^ – hypothalamic H/O neurons are thought to play a role in setting neuroendocrine output. Our data, demonstrating that H/O can modulate the neuroendocrine system that controls pituitary Prl release, underscores this contention, demonstrating that H/O neurons are an important regulatory node for hypothalamic-pituitary interaction.

The H/O-ergic system is a key element of the neurocircuitry orchestrating biorhythms^58^. Our data provide a compelling cellular and molecular mechanism by which H/O peptides can influence the daily rhythm of Prl secretion, which is lowest during waking when release of H/Os are at their peak^59^,^60^ and their positive influence on TIDA activity and dopamine secretion (which exhibits a similar circadian pattern^14^) may be at its greatest. Furthermore, our data demonstrate that this influence occurs post-synaptically, with H/Os directly exciting TIDA neurons in a cell-autonomous fashion. This H/O induced excitation of TIDA neurons is of particular interest as previous data has suggested that part of H/O’s effect upon Prl secretion may be independent of hypothalamic dopamine, acting via the NPYergic system^23^,^61^.

The direct, postsynaptic effects of H/O on TIDA neurons shown here may be a physiological correlate of the heavy innervation of the Arc – where TIDA neurons are located^62^ – by H/O-immunoreactive fibers^17^,^33^. Indeed, we demonstrate that H/O-ir terminals form close appositions onto TIDA cell bodies and proximal dendrites, indicative of synaptic contacts. This evidence, in conjunction with our electrophysiological data, suggest that the powerful Prl-releasing actions of centrally administered H/O^22^ are mediated through the TIDA system. As H/O fibers are present in the median eminence and the OX1R receptor is abundantly expressed in (as yet unidentified) pituitary cells^63^,^64^, parallel actions at the level of the pituitary cannot be excluded. Despite this, conclusive evidence for a pituitary effect of H/O on Prl secretion is lacking; in the rat no such actions are seen^65^; while in the sheep, only modest, seasonally dependent effects have been shown^66^. Notably, in our immunofluorescence stainings H/O-ir terminals in the median eminence were few in number and substantially lower in density compared to those in the dmArc.

In addition to H/O, the pineal hormone melatonin is another chronobiological factor thought to regulate pituitary Prl secretion. Previous research has indicated that melatonin’s Prl-releasing effects may be independent of dopamine neurosecretion^67^,^68^,^69^, yet whether melatonin directly modulates the electrical properties of TIDA neurons has not been addressed. We found that melatonin application failed to alter either the oscillatory activity or the membrane potential of TIDA neurons. This lack of response further supports the notion that melatonin exerts its influence upon Prl secretion at a site other than the TIDA network, most likely through the pituitary pars tuberalis (see ref. 45).

Beyond the potential implications for our understanding of the physiological nychthemeral release of Prl, our results are interesting in the context of clinical reports indicating that some narcolepsy patients – who show markedly reduced numbers of H/Oergic cells^18^,^19^ – exhibit elevated serum Prl^70^,^71^. In this scenario the absence of a wakefulness-correlated stimulatory influence (*i.e.* H/O) on TIDA neurons could contribute to relieving pituitary lactotrophs from their dopaminergic “brake” resulting in the hyperprolactinaemia observed. It should be noted, however, that other investigators have failed to observe changes in circulating Prl in narcoleptic patients^72^,^73^,^74^.

While this clinical question remains to be resolved, the present data support a role for the hypothalamic H/Oergic system in the control of neuroendocrine and anterior pituitary function. The excitation of TIDA neurons shown here may be a part of the explanation for the circadian secretion pattern of Prl. Future investigations will determine how H/O input coordinates with other candidate circadian influences on TIDA neurons, including the suprachiasmatic nucleus^13^ and the expression of clock genes in the dopamine neurons themselves^14^.

## Additional Information

**How to cite this article:** Lyons, D. J. *et al*. Hypocretin/Orexin Peptides Excite Rat Neuroendocrine Dopamine Neurons through Orexin 2 Receptor-Mediated Activation of a Mixed Cation Current. *Sci. Rep.* **7**, 41535; doi: 10.1038/srep41535 (2017).

**Publisher's note:** Springer Nature remains neutral with regard to jurisdictional claims in published maps and institutional affiliations.
